# AQUACEL® Ag Surgical Dressing Reduces Surgical Site Infection and Improves Patient Satisfaction in Minimally Invasive Total Knee Arthroplasty: A Prospective, Randomized, Controlled Study

**DOI:** 10.1155/2017/1262108

**Published:** 2017-08-02

**Authors:** Feng-Chih Kuo, Bradley Chen, Mel S. Lee, Shih-Hsiang Yen, Jun-Wen Wang

**Affiliations:** ^1^Department of Orthopedic Surgery, Kaohsiung Chang Gung Memorial Hospital and Chang Gung University, College of Medicine, Kaohsiung, Taiwan; ^2^Institution of Public Health, National Yang-Ming University, Taipei, Taiwan

## Abstract

The use of modern surgical dressings to prevent wound complications and surgical site infection (SSI) after minimally invasive total knee arthroplasty (MIS-TKA) is lacking. In a prospective, randomized, controlled study, 240 patients were randomized to receive either AQUACEL Ag Surgical dressing (study group) or a standard dressing (control group) after MIS-TKA. The primary outcome was wound complication (SSI and blister). The secondary outcomes were wear time and number of dressing changes in the hospital and patient satisfaction (pain, comfort, and ease of use). In the intention-to-treat analysis, there was a significant reduction in the incidence of superficial SSI (0.8%, 95% CI∶ 0.00–2.48) in the study group compared to 8.3% (95% CI∶ 3.32–13.3) in the control group (*p* = 0.01). There were no differences in blister and deep/organ-space SSIs between the two groups. Multivariate analysis revealed that AQUACEL Ag Surgical dressing was an independent risk factor for reduction of SSI (odds ratio: 0.07, 95% CI: 0.01–0.58, *p* = 0.01). The study group had longer wear time (5.2 ± 0.7 versus 1.7 ± 0.4 days, *p* < 0.0001) and lower number of dressing changes (1.0 ± 0.2 versus 3.6 ± 1.3 times, *p* < 0.0001). Increased patient satisfaction (*p* < 0.0001) was also noted in the study group. AQUACEL Ag Surgical dressing is an ideal dressing to provide wound care efficacy, patient satisfaction, reduction of SSI, and cost-effectiveness following MIS-TKA.

## 1. Introduction

Periprosthetic joint infection (PJI) is a severe complication and occurs in 1-2% of patients after total knee arthroplasty (TKA) [1]. Recently, a study of primary causes of revision TKA found that PJI comprised 14.5% of total revision and 26.8% of cases if revision was performed within one year after index operation [2]. One risk factor related to PJI is superficial wound complication, including surgical site infection (SSI), prolonged wound discharge, and skin blisters [3]. Therefore, prevention of superficial wound complications is necessary after TKA.

Minimally invasive surgery (MIS) has gained popularity in TKA with the advantages of shortened wound length, decreased rehabilitation period, and quicker return to work compared to standard TKA [4]. MIS-TKA also has higher wound complications, which are related to greater tension on wound edges during surgery [5]. Therefore, an improved wound care modality is essential. In our institution, the standard dressing care after MIS-TKA is an antimicrobial dressing (Sofra-Tulle®, Royal Chem. & Pharm. Co., Ltd., Kaohsiung, Taiwan) on the inner layer and gauzes with tape on the outer layer. However, patients often complained of pain during dressing change and discomfort during knee range-of-motion exercise after surgery by the use of gauze dressings [6]. Furthermore, skin blistering and infection are common problems because postoperative movement around the knee joint causes friction between the skin and traditional gauze [7].

AQUACEL Ag Surgical dressing (ConvaTec Inc., Greensboro, North Carolina, USA) is a modern dressing. The dressing comprises a core hydrofiber layer containing ionic silver that absorbs exudates to form a cohesive gel and provides antimicrobial protection and an adhesive hydrocolloid backing that fully protects the wound. Both hydrofiber and hydrocolloid layers are extensible to accommodate skin movement during postoperative physiotherapy and prevent blistering [9]. Few comparisons of the AQUACEL Ag Surgical dressing and the standard dressing have been reported on traditional TKA [11–13], and the literature on MIS-TKA is sparse.

We hypothesized that AQUACEL Ag Surgical dressing would have a significant improvement in the efficacy of wound care, patient satisfaction, and surgical site infection compared with standard dressings after MIS-TKA.

## 2. Materials and Methods

**Figure 1:**
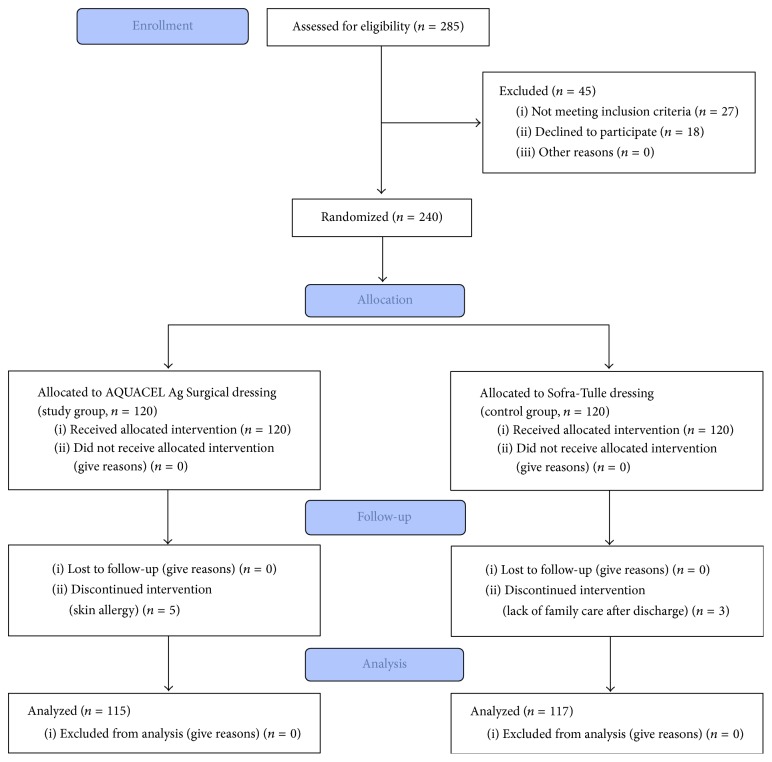
CONSORT flow diagram showing enrollment and exclusion through the trial phase.

A prospective, randomized, controlled trial was conducted involving a consecutive series of patients undergoing primary MIS-TKA at a single institute between October 2013 and September 2014. Written informed consent was obtained from all patients before their participation in the study. The present study was approved by the institutional review board of our institution and was registered in the public ClinicalTrials.gov registry (NCT02445300). All patients were enrolled in accordance with the Consolidated Standards of Reporting Trials (CONSORT) (Figure 1).

Inclusion criteria included the patients who were scheduled for primary unilateral MIS-TKA in the study period. The indication for TKA was severe osteoarthritis of the knee. Exclusion criteria included patients with condition or comorbidity that could compromise wound healing, including varicose vein, peripheral vascular disease, smokers, poor nutrition, receiving immunosuppressive medications, corticosteroid abuse, and chronic skin disease around the knee (e.g., psoriasis and chronic eczema). Patients who* had* had prior knee replacement, an osteotomy, or a fracture of the ipsilateral knee were also excluded. Therefore, 285 patients were enrolled. Twenty-seven patients were further excluded due to the condition or comorbidity that could compromise wound healing. Eighteen patients who declined to participate were also excluded from the study. Finally, 240 patients were randomized to receive either AQUACEL Ag Surgical dressing (study group) or Sofra-Tulle dressing (control group) after MIS-TKA. A computer-generated randomization schedule was used to assign participants to treatment using a block size of 8 (1 : 1 ratio) (Figure 1).

Before the study, 240 opaque sealed envelopes were numbered randomly from 1 to 240 by means of a computer-generated method: 120 envelopes containing 3 pieces of AQUACEL Ag Surgical dressing (9 cm × 25 cm) and 120 envelopes containing 10 pieces of Sofra-Tulle dressings (10 cm × 10 cm). All patients received unilateral primary MIS-TKA under general anesthesia. A pneumatic thigh tourniquet was inflated to a pressure of 300 mmHg before the incision and deflated at the end of surgery after skin closure. All wounds were closed with interrupted skin stitches.

All patients received minimally invasive surgery by the same surgeon. All TKAs were cemented using the same type of prosthesis (NexGen, Legacy, Posterior-Stabilized Prosthesis; Zimmer, Warsaw, IN). A mini-midvastus approach for TKA was employed, as described by Haas et al. [14]. The skin incision was made along the medial aspect of the patella to the medial border of the mid-to-distal tibial tubercle. The patellar components were all resurfaced. There was no local infiltration of local anesthetic. A suction drain was inserted at the end of the operation and was removed two days after the operation. At the end of skin closure, a sealed envelope was opened to notify the surgeon of the closure method. The dressing was applied to the wound in the operating theater by the surgeon. All patients received oral Factor Xa inhibitor as deep vein thrombosis prophylaxis for 14 days. A standard postoperative rehabilitation protocol was applied to all patients, including the use of continuous passive motion of the knee and muscle strengthening exercise immediately after surgery. All patients were taught by a physical therapist to get out of bed with walker support on the first postoperative day.

The study group used AQUACEL Ag Surgical dressing. The indications for removal of the AQUACEL Ag Surgical dressing were leakage beyond the hydrocolloid exterior layer and more than 50% saturation of the hydrofiber inner layer [9]. If there were no indications to change the dressing, it was changed at the day of discharge, usually the 4th or 5th postoperative day (POD), and the wound remained covered for 7 days except for exudates across the dressing. Then, a new AQUACEL Ag Surgical dressing was applied at home until the first visit at the clinic. The control group used Sofra-Tulle dressing, which is an antimicrobial dressing formed by a fabric of leno weave impregnated with white soft paraffin containing 1% framycetin sulphate. The standard dressing consisted of a Sofra-Tulle dressing on the inner layer covered with gauze on the outer layer and was occlusive with tapes over the whole surface of the standard dressing. The indication for removal of the standard dressing was wound drainage on the dressing. If the wound was not soiled, it was changed on the day of discharge. After being discharged from the hospital, the family conducted the dressing change according to the removal criteria for each dressing.

### 2.1. Outcomes Measurements

The primary outcome measure was wound complication, including surgical site infection (SSI) and blister. Wound complication was assessed* at* each dressing change. SSI was defined based on the recent recommendations of the Centers for Disease Control and Prevention (CDC) and divided into superficial SSI (only involving skin and subcutaneous tissue) within 3 months after surgery, deep SSI (involving below the fascia), and organ-space SSI (involving the joint) within 1 year after surgery [15]. The secondary outcome was patient satisfaction about the dressings. Patient satisfaction was evaluated by three parameters (pain, comfort, and ease of use) on the day of the first postoperative visit. Pain was evaluated with the use of a visual analog scale (VAS), with 0 representing “no pain” and 10 representing “severe pain” [16]. The pain severity was reported by the patient during dressing removal. The comfort and ease of use were classified as excellent, good, fair, or poor [17]. Wear time of the dressing and number of dressing changes in the hospital were also recorded. All the patients completed the outcome evaluation.

### 2.2. Statistical Analysis

An a priori sample size was estimated using a 2-tailed Fisher exact test with a 0.05 level of significance. Based on the study conducted by Burke and colleagues [9], we estimated the incidence of wound complication at 4.8% in the study group and 17.7% in the control group. We determined that 204 participants (102 per group) would be needed to achieve 80% statistic power. Expecting a 15% attrition rate, a total of 240 patients were enrolled (120 per group).

The categorical data were summarized as an absolute value and percentage. The continuous data were presented as mean and standard deviation. Independent samples* t*-test was used to compare the continuous variables and the chi-squares test was used to compare the categorical variables. Wound complication rates and patient satisfaction were expressed by calculation of proportion and a 95% confidence interval (CI). The primary prespecified analysis was an intention-to-treat analysis. The intention-to-treat population included all 240 patients who underwent randomization. We also performed a prespecified per-protocol analysis. The per-protocol population included patients in both groups who had used the same dressings throughout the study. Multivariate logistic regression was used to determine whether AQUACEL Ag Surgical dressing was an independent predictor for surgical site infection. The multivariate logistic regression incorporated the following demographics: age, sex, BMI, ASA, and comorbidities. A 5% statistically significant level was prescribed (*p* < 0.05). All data were analyzed with the use of MedCalc software (version 17.4, Ostend, Belgium).

## 3. Results

A total of 240 patients underwent randomization (Figure 1). Five patients had skin allergies after application of AQUACEL Ag Surgical dressing and were switched to standard dressing. Three patients in the control group refused to participate in the study after allocation due to the lack of family care after discharge and then switched to use AQUACEL Ag Surgical dressing. All 240 patients were included in the intention-to-treat analysis, whereas 115 of 120 patients (95.8%) in the study group and 117 of 120 patients (97.5%) in the control group were included in the per-protocol analysis. No patients were lost during two-year follow-up.

The basic demographic data were similar between the two groups (Table 1). The length of hospital stay did not differ significantly between the two groups (6.3 ± 1.1 versus 6.6 ± 1.4 days, *p* = 0.02). Patients in the study group had a longer mean wear time (5.2 ± 0.7 days) than those in the control group (1.7 ± 0.4 days, *p* < 0.0001). The mean number of dressing changes prior to discharge was significantly lower in the study group (1.0 ± 0.2 times) than in the control group (3.6 ± 1.3 times, *p* < 0.0001).

Of the eight dropouts, only one patient developed blisters in the study group. None of these dropouts developed surgical site infection. In the intention-to-treat analysis, the incidence of blistering was lower in the study group at 2.5% (3/120, 95% CI: 0.00–5.33) compared to 5.0% (6/120, 95% CI: 1.04–8.96) in the control group (*p* = 0.31). The incidence of superficial SSI in the study group was statistically significantly lower at 0.8% (1 of 120, 95% CI: 0.00–2.48) compared to 8.3% (10 of 120, 95% CI: 3.32–13.3) in the control group (*p* = 0.01). One patient developed deep SSI in the control group (0.8%, 95% CI: 0.00–2.48), but no patients had deep or organ-space SSI in the study group (*p* = 0.32) (Table 2). The multivariate logistic regression revealed that AQUACEL Ag Surgical dressing was an independent risk factor for PJI with an odds ratio (OR) of 0.07 (95% CI: 0.01–0.58, *p* = 0.01).

The patient satisfaction is shown in Table 3. The mean VAS pain score was lower in the study group compared with the control group when the dressing was removed (1.1 ± 0.7 versus 3.6 ± 1.2, *p* < 0.0001). In the study group, most patients experienced excellent comfort when the dressing was in place (67.8% versus 31.6%, *p* < 0.0001) and during removal (74.8% versus 42.7%, *p* < 0.0001). Excellent ease of use was rated higher in the study group compared with the control group during application of the dressing (92.2% versus 35.0%, *p* < 0.0001) and removal of the dressing (95.7% versus 40.2%, *p* < 0.0001).

## 4. Discussion

AQUACEL with or without silver-impregnated dressing has been shown to be an effective dressing to significantly reduce the occurrence of acute PJI [11], blister formation [10], and SSI [8, 9] after total joint arthroplasty compared to other adhesive dressings in previous studies (Table 4). In a case-control study by Cai et al. [11], the incidence of PJI was lower in the AQUACEL Ag Surgical dressing group compared to the standard gauze dressing group (0.44% versus 1.7%, *p* = 0.005). They found that the use of AQUACEL Ag Surgical dressing was an independent risk factor for reduction of PJI (OR: 0.17, 95% CI: 0.05–0.53). Dobbelaere et al. [12] compared three innovative wound dressings to each other and to a standard dressing after total knee arthroplasty. The innovative wound dressings were Opsite Post-Op Visible® (Smith & Nephew Advanced Wound Management, Hull, UK), AQUACEL Surgical®, and Mepilex® Border (Mölnlycke Health Care, Gothenburg, Sweden). The standard wound dressings were Zetuvit® (Paul Hartmann AG, Heidenheim, Germany), immediately applied after TKA, followed on the first postoperative day by Cosmopor® E (Paul Hartmann AG, Heidenheim, Germany). They found no infection in all patients with the use of these three innovative wound dressings. Springer et al. [13] also reported 0% SSI with the use of AQUACEL Ag Surgical dressings in total hip and knee arthroplasty, but they could not conclude that AQUACEL Ag Surgical dressings played an important role in reducing SSI. Our study agreed that AQUACEL Ag Surgical dressing is an independent risk factor for reduction of SSI following MIS-TKA (OR: 0.07, 95% CI: 0.01–0.58). The silver-containing dressing has been proven to fight against commonly encountered wound pathogens, including antibiotic-resistant bacteria such as methicillin-resistant* Staphylococcus aureus* and vancomycin-resistant enterococci, aerobic and anaerobic bacteria, and yeasts in an in vitro study [18]. Upon hydration of exudates, the hydrofiber dressing responds to changes in wound fluid and silver ions are continuously made available during dressing wear time, which reduced SSI in the clinical study. However, we did not find differences in blister formation between the two types of dressings. We considered two reasons. First, our cases were performed using minimally invasive surgery, which avoided eversion of the patella and dissection of the lateral skin flap of the knee [14]. Therefore, the blood supply of the skin flap around the knee may be less compromised. Second, the standard dressing used in our study has nonadherent properties. In previous reports comparing modern and traditional dressings [8–10], the traditional dressing was an adhesive dressing (Mepore®; Mölnlycke Health Care, Norcross, Georgia), which caused increased skin blister formation compared to nonadherent dressings [19].

Our study also showed that AQUACEL Ag Surgical dressing had increased patient satisfaction in terms of pain, comfort, and ease of use compared to standard of care. However, five patients in the study group dropped out of the study because they had skin itching and erythema after application of the AQUACEL Ag Surgical dressings. According to Dobbelaere et al. [12], skin irritation and redness were not found in the AQUACEL Ag Surgical group, but 12.9% of the patients experienced these reactions in the conventional dressing group. They also found that AQUACEL Ag Surgical dressing had better scores for pain, freedom of movement, and general comfort compared to the conventional dressing. In a prospective randomized clinical trial, hydrofiber dressing with ionic silver was better for managing pain, overall comfort, wound trauma upon dressing removal, exudate handling, and ease of use compared to povidone-iodine gauze for the treatment of open surgical and traumatic wounds [17]. Similar results were reported when hydrofiber dressing was applied for chronic leg ulcerations [20]. The reasons for better patient satisfaction in AQUACEL Ag Surgical dressing are attributed to the hydrofiber layer and hydrocolloid layer. The individual fibers in hydrofiber dressings are fine and flexible. The hydrocolloid layer is skin-friendly and comfortable during body movement [21]. With those two characteristics, the AQUACEL Ag Surgical dressing is extensible to accommodate skin movement during physiotherapy and that is associated with reduced blistering after TKA.

The cost of one AQUACEL Ag Surgical dressing at our institution is US$15. A standard taped gauze dressing costs nearly US$1. Therefore, the additional cost for an AQUACEL Ag Surgical dressing is about US$14 per case. In Taiwan, there are approximately 25 thousand TKAs performed annually. The cost of using an AQUACEL Ag Surgical dressing routinely after TKA would add approximately* US$350,000* in cost. Infection after TKA has been reported with an incidence ranging from 1.0% to 2.0% [1]. The cost to treat a PJI has been estimated to range from* US$13,000* to over* US$23,000* in Taiwan [22]. In the Taiwan* study*, the annual low-end cost for the treatment of PJI would be* US$3.25 million* assuming the lower incidence of reported PJI and lower cost of PJI treatment. If the reported thirteenfold reduction in SSI noted in our study is correct, the cost saving would be reduced to* US$3 million* with the use of an AQUACEL Ag Surgical dressing compared to the control dressing in PJI management using the lower estimate. In the United States, the use of AQUACEL Ag Surgical dressing can result in fourfold reduction in SSI, and thus the cost of PJI management would be reduced at approximately* US$375 million* [11].

We have acknowledged some limitations in this study. First off, allocation concealment was performed using opaque envelopes. However, the differences in the dressing sizes would most certainly mean that those involved in administering the intervention dressings were not blinded and would be aware of upcoming assignments. Moreover, patients were not capable of comparing the 2 dressings when ranking satisfaction. Second, our control group used Sofra-Tulle dressing, which has improved characteristics in wound care such as nonadherent properties and antimicrobial effects rather than simple gauzes accompanied with tapes. Third, the patient's family conducted the wound care after discharge from the hospital. Wound complications may increase if inadvertent wound care is performed. In addition, the indications for early dressing change in both groups were somewhat subjective and there should be a selection bias in the evaluation of the number of dressing changes. Finally, the patient satisfaction assessment did not use a validated tool and patients were not able to compare the two dressings directly.

## 5. Conclusion

Our prospective, randomized, controlled trial demonstrated that the use of AQUACEL Ag Surgical dressing contributes* favorably* to both the clinical efficacy and the cost-effectiveness for managing wound care that is associated with minimally invasive total knee arthroplasty.

## Figures and Tables

**Table 1:** Demographics of the patients.

	AQUACEL Ag Surgical (study dressing)	Sofra-Tulle (control dressing)	*p* value
Age (years), mean ± SD	70.3 ± 7.5	70.1 ± 7.1	0.85
BMI (kg/m^2^), mean ± SD	27.8 ± 4.6	27.7 ± 4.4	0.86
Sex F/M, *n*	85/35	91/29	0.38
ASA, *n* (%)			0.19
I	12 (10.0)	10 (8.3)	
II	65 (54.2)	53 (44.2)	
III	43 (35.8)	57 (46.7)	
Diabetic, *n* (%)	23 (19.2)	18 (15.0)	0.39
Chronic kidney disease, *n* (%)	9 (7.5)	11 (9.2)	0.64
Cardiovascular, *n* (%)	17 (14.2)	14 (11.7)	0.56

SD: standard deviation; BMI: body mass index; F: female; M: male; ASA: American Society of Anesthesiologists.

**Table 2:** Surgical site infection and blistering estimated according to intention-to-treat and per-protocol analysis in patients treated with AQUACEL Ag Surgical dressing and control dressing.

Wound complications	AQUACEL Ag Surgical (study dressing) (dropouts, *n* = 5)		Sofra-Tulle (control dressing) (dropouts, *n* = 3)	
	ITT analysis	PP analysis	ITT analysis	PP analysis
Blistering, **%** (95**%** CI)	2.5 (0.00–5.33)	1.7 (0.00–4.17)	5.0 (1.04–8.96)	5.1 (1.07–9.18)
Superficial SSI, **%** (95**%** CI)	0.8 (0.00–2.48)	0.9 (0.00–2.59)	8.3 (3.32–13.3)	8.5 (3.41–13.7)
Deep/organ-space SSI, **%** (95**%** CI)	0	0	0.8 (0.00–2.48)	0.9 (0.00–2.55)

SSI: surgical site infection; CI: confidence interval; 95% CI: 95% confidence interval; ITT: intention-to-treat; PP: per-protocol.

**Table 3:** Patient satisfaction.

	AQUACEL Ag Surgical (study dressing)	Sofra-Tulle (control dressing)	*p* value
Pain (VAS)			
Dressing removal, mean ± SD	1.1 ± 0.7	3.6 ± 1.2	<0.0001
Comfort (excellent), **%** (95% CI)			
Dressing in place	67.8 (59.1–76.5)	31.6 (23.0–40.2)	<0.0001
Dressing removal	74.8 (66.7–82.8)	42.7 (33.6–51.8)	<0.0001
Ease of use (excellent), **%** (95% CI)			
Ease of application	92.2 (87.2–97.2)	35.0 (21.5–38.3)	<0.0001
Ease of removal	95.7 (91.9–99.4)	40.2 (31.2–49.2)	<0.0001

VAS: visual analog scale; SD: standard deviation; 95% CI: 95% confidence interval.

**Table 4:** Comparison of reported literatures on AQUACEL with or without silver-impregnated dressing following total joint arthroplasty.

Study	Design	Dressing	Number of patients	Surgery	Wear time (days)	Dressing change (number)	Blister (%)	SSI (%)
Clarke et al. (2009) [8]	Prospective	Folded AQUACEL and hydrocolloid dressing	242	TKA and THA	3.7	1.5	2	1
Burke et al. (2012) [9]	Prospective randomized	AQUACEL and hydrocolloid dressing	62	27 TKAs 35 THAs	—	1	4.8	0
Hopper et al. (2012) [10]	Prospective	AQUACEL Surgical	50	25 TKAs 24 THAs 1 RTHA	7	0	4	4
Cai et al. (2014) [11]	Case-controlled	AQUACEL Ag Surgical	903	508 TKAs 392 THAs 3 TKAs + THAs	—	—	—	0.4
Dobbelaere et al. (2015) [12]	Prospective randomized	AQUACEL Ag Surgical	29	29 TKAs	—	0.67	6.9	0
Springer et al. (2015) [13]	Prospective randomized	AQUACEL Ag Surgical	141	74 TKAs 67 THAs	—	0.14	0.7	0
This study	Prospective randomized	AQUACEL Ag Surgical	115	115 MIS-TKAs	5.2	1	1.7	0.8

TKA: total knee arthroplasty; THA: total hip arthroplasty; RTHA: revision total hip arthroplasty; SSI: surgical site infection.
